# Alternative Base Solutions for del Nido Cardioplegia: Composition, Rationale, and Clinical Evidence

**DOI:** 10.1093/icvts/ivag019

**Published:** 2026-01-13

**Authors:** Narongrit Kantathut

**Affiliations:** Division of Thoracic and Cardiovascular Surgery, Department of Surgery, Ramathibodi Hospital, Mahidol University, Bangkok 10400, Thailand

**Keywords:** del Nido cardioplegia, modification, alternative base solution, myocardial protection, cardiac surgery

## Abstract

**Objectives:**

Del Nido cardioplegia has gained increasing popularity, evolving from its original use in paediatric and congenital heart surgery to widespread adoption in adult cardiac procedures. This review aims to summarize the composition, physiologic rationale, and clinical evidence supporting the use of alternative base solutions in del Nido cardioplegia.

**Methods:**

A comprehensive literature review was conducted, analyzing experimental, observational, and randomized clinical studies that compared modified del Nido cardioplegia prepared with various alternative crystalloids.

**Results:**

Evidence demonstrates that del Nido cardioplegia prepared with alternative crystalloids including normal saline, lactated Ringer’s, plain Ringer’s, Isolyte S, Ionosteril, and whole blood provides myocardial protection and postoperative outcomes comparable with Plasma-Lyte A when appropriately buffered and supplemented.

**Conclusions:**

Alternative base solutions including normal saline, lactated Ringer’s, plain Ringer’s, Isolyte S, Ionosteril, and whole blood represent safe, effective, and cost-efficient substitutes for Plasma-Lyte A in del Nido cardioplegia. Standardized formulations and multicentre randomized studies are warranted to confirm long-term safety and support broader global implementation.

## Graphical abstract

**Figure:**
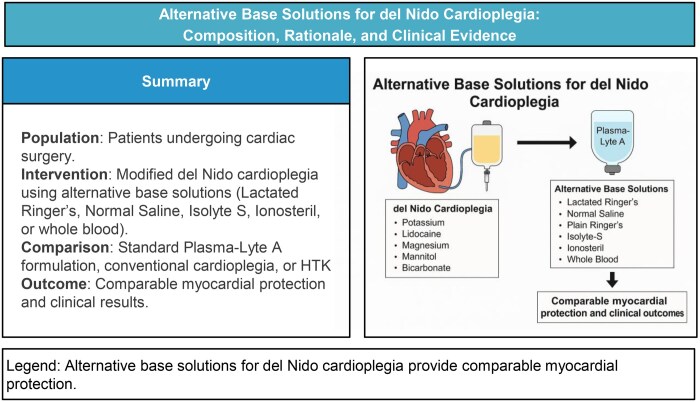

## INTRODUCTION

Myocardial protection is a critical determinant of success in cardiac surgery. Among the various cardioplegia strategies, *del Nido cardioplegia* has emerged as one of the most widely used solutions. It was originally developed in the early 1990s by Dr Pedro J. del Nido and colleagues at the University of Pittsburgh for use in paediatric and congenital heart surgery, with the goal of achieving prolonged myocardial protection with a single-dose strategy. The formulation was subsequently adopted and used extensively at Boston Children’s Hospital, where it demonstrated excellent safety and efficacy over nearly 2 decades of continuous clinical experience.^1^^,^^2^ Over time, its effectiveness, simplicity, and safety have led to widespread adoption in adult cardiac surgery.^3–6^ The key advantages of the del Nido strategy include its single-dose delivery, reliable myocardial protection during prolonged ischaemic periods, and reduction in operative interruptions for re-dosing.

The original del Nido formulation employs Plasma-Lyte A (Baxter Healthcare, Deerfield, Illinois) as the crystalloid base, combined with potassium chloride, lidocaine, magnesium sulfate, mannitol, and sodium bicarbonate. This composition was designed to achieve rapid depolarized arrest, stabilize cellular membranes, buffer acidosis, mitigate reperfusion injury, and reduce oxidative stress. Plasma-Lyte A was specifically chosen for its physiologic electrolyte composition, absence of calcium, and buffering capacity through acetate and gluconate.

Despite these advantages, Plasma-Lyte A is not universally available and is relatively costly compared with other intravenous fluids. These limitations have prompted the exploration of alternative base solutions—including lactated Ringer’s, normal saline, and other balanced crystalloids—for the preparation of del Nido cardioplegia. Each alternative differs in sodium, chloride, potassium, calcium, buffering agents, and pH, factors that may influence myocardial physiology during ischaemia and reperfusion. Over the past decade, clinical experiences and randomized studies have begun to evaluate these alternatives, with emerging evidence suggesting that some may provide comparable outcomes to Plasma-Lyte A in both adult and paediatric cardiac surgery.

This review provides a comprehensive overview of alternative base solutions for del Nido cardioplegia, focusing on their composition, physiologic rationale, experimental and clinical evidence, and practical considerations. By consolidating available data, we aim to clarify whether these alternatives can be safely adopted into routine practice and to identify key gaps that warrant future investigation.

## COMPOSITION OF DEL NIDO CARDIOPLEGIA

The original del Nido cardioplegia solution is prepared using Plasma-Lyte A as the crystalloid base, to which several pharmacologic additives are incorporated to achieve myocardial arrest and protection. Plasma-Lyte A provides a physiologic electrolyte profile that closely resembles human plasma, containing sodium, chloride, potassium, magnesium, acetate, and gluconate, with a near-physiologic pH of 7.4 and an osmolarity of approximately 294 mOsm/L (Table 1). To this base, the following additives are mixed into 1000 mL of the crystalloid component: potassium chloride 13 mL (2 mEq/mL), lidocaine 13 mL (1%), magnesium sulfate 4 mL (50%), mannitol 16.3 mL (20%), and sodium bicarbonate 13 mL (8.4%, 1 mEq/mL), resulting in a final solution that is intentionally calcium-free. Each component serves a distinct role: potassium induces rapid depolarized arrest; lidocaine blocks sodium channels, thereby prolonging arrest and stabilizing myocardial membranes; magnesium functions as a calcium antagonist, protecting against calcium overload during reperfusion; mannitol acts as both an osmotic agent to reduce cellular oedema and a free-radical scavenger; and sodium bicarbonate provides buffering against acidosis. Together, these elements create a solution that promotes prolonged electrical quiescence and metabolic support, enabling effective myocardial protection with a single-dose strategy that is particularly advantageous in complex or extended surgical procedures.^1^^,^^2^

**Table 1.** Composition of Base Solutions

Component (mEq/L)	Plasma	PlasmaLyte-A	Normal saline	Lactated Ringer	Acetated Ringer	Plain Ringer	Isolyte S	Ionosteril
Sodium (Na^+^)	135-140	140	154	130	130	147	141	137
Chloride (Cl^−^)	95-105	98	154	109	108.7	155	98	110
Potassium (K^+^)	3.5-5.3	5	0	4	4	4	5	4
Magnesium (Mg²^+^)	1.6-2.4	3	0	0	0	0	3	2.5
Calcium (Ca²^+^)	4.4-5.2	0	0	2.7	2.7	4	0	3.3
HCO_3_^−^	24-32	0	0	0	0	0	0	0
Buffers	Protein, Phosphate (1-2), Hb^a^	Acetate (27) Gluconate (23)	–	Lactate 27.7	Acetate 28	–	Acetate (27) Gluconate (23) Phosphate (1)	Acetate (36.8)
Osmolality (mOsm/L)	275-295	294	308	273.4	273	311.3	295	291
pH	7.35-7.4	∼7.4 (6.5-8.0)	∼5.5 (4.5-7.0)	∼6.5-7.5	6.5-8.0	5.8	∼7.4 (7.0-7.8)	5.0-7.0

a Hb, hemoglobin (present only in whole blood).

## NORMAL SALINE

Normal saline (NSS, 0.9% sodium chloride) is one of the most widely available intravenous fluids worldwide. It contains sodium 154 mmol/L and chloride 154 mmol/L, has a pH of approximately 5.5, and an osmolarity of 308 mOsm/L, making it slightly hypertonic compared with plasma (Table 1). Its simplicity, universal availability, and low cost make it an attractive potential base solution in resource-limited settings where Plasma-Lyte A is not accessible. However, several limitations reduce its suitability as a base for del Nido cardioplegia. Unlike Plasma-Lyte A or other balanced crystalloids, normal saline lacks potassium, magnesium, buffer agents, and organic anions such as acetate or gluconate. Its high chloride concentration predisposes patients to hyperchloremic metabolic acidosis, particularly in the context of cardiopulmonary bypass where large volumes may be administered. In addition, its slightly hypertonic nature can draw water out of myocardial cells, resulting in cellular shrinkage, which may adversely affect myocardial metabolism and function. Moreover, the low pH and absence of buffering capacity may exacerbate systemic and myocardial acidosis during ischaemia and reperfusion.

Evidence supporting the use of normal saline as a feasible base solution for del Nido cardioplegia has evolved progressively through experimental and clinical studies over the past decade. The concept was first validated by Nakao et al in a controlled piglet ischaemia-reperfusion model, which compared saline-based modified del Nido cardioplegia with the original Plasma-Lyte A formulation. The authors demonstrated equivalent myocardial functional recovery, comparable histologic preservation with reduced oedema, and no adverse metabolic effects, confirming that NSS can safely serve as a carrier solution without compromising myocardial protection.^7^

Building on this foundation, Sevuk et al conducted a propensity-matched study in 200 adult cardiac surgical patients, comparing tepid NSS-based modified del Nido cardioplegia (4:1 blood-to-crystalloid ratio, 28°C) with conventional cold blood cardioplegia. The results showed no significant differences in troponin T release, inotrope requirement, defibrillation need, or postoperative ventricular function, while the NSS-based strategy yielded shorter cardiopulmonary bypass (CPB), aortic cross-clamp, and total operative times, supporting its safety and procedural efficiency.^8^

Expanding this evidence, Seleem et al performed a prospective randomized controlled trial in 80 adults undergoing mitral valve surgery, comparing NSS-based modified del Nido cardioplegia with traditional St Thomas cardioplegia. The NSS-based group exhibited lower CK-MB, troponin T, and lactate levels; higher postoperative ejection fractions; and reduced transfusion needs, indicating superior biochemical myocardial protection without increasing complications or mortality.^9^

Mostafa et al evaluated NSS-based modified del Nido cardioplegia in 46 adults with low ejection fraction undergoing combined valve and coronary bypass grafting (CABG) procedures; the authors reported shorter CPB and cross-clamp times, fewer re-doses, lower inotrope use, shorter intensive-care-unit (ICU) stay, faster extubation, and significantly lower troponin T and CK-MB at 24 hours—demonstrating greater efficiency and myocardial preservation in high-risk patients.^10^

Ali et al conducted a double-blind randomized clinical trial involving 120 adult CABG patients, comparing NSS-based, Ringer lactate-based, and plain Ringer-based modified del Nido cardioplegia. All groups achieved comparable myocardial protection, with no significant differences in biochemical or clinical outcomes, confirming that NSS provides equivalent efficacy to balanced crystalloids.^11^

Finally, Kurogochi et al provided veterinary clinical validation in a randomized controlled trial of 40 dogs undergoing mitral valve repair, comparing saline-based modified del Nido cardioplegia with St Thomas cardioplegia. The NSS-based group showed no significant difference in troponin I levels but demonstrated faster recovery of sinus rhythm and similar long-term survival and ventricular remodelling, reinforcing its translational applicability and supporting the effectiveness of saline-based formulations for myocardial protection.^12^

Cumulative evidence from experimental, clinical, and veterinary studies consistently supports the safety and efficacy of NSS as an alternative base solution for del Nido cardioplegia. Across diverse models—from animal experiments to randomized human trials—NSS-based modified del Nido cardioplegia has demonstrated comparable myocardial protection to formulations prepared with balanced crystalloids such as Plasma-Lyte A or Ringer’s solutions, with added benefits of simplicity, accessibility, and cost-effectiveness. Although concerns remain regarding its lack of buffering capacity and potential for hyperchloremic acidosis, clinical outcomes have not shown adverse metabolic or functional effects when appropriately buffered with bicarbonate. These findings highlight NSS-based del Nido cardioplegia as a practical, effective, and globally applicable option, particularly valuable for resource-limited cardiac centres where Plasma-Lyte A is unavailable.

## LACTATED RINGER’S SOLUTION

Lactated Ringer’s solution (LRS) is widely available, inexpensive, and commonly used for fluid resuscitation. Its composition includes sodium 130 mmol/L, chloride 109 mmol/L, potassium 4 mmol/L, calcium 1.5 mmol/L, and lactate 28 mmol/L, with a pH of approximately 6.5 and an osmolarity of 274 mOsm/L, which is slightly hypotonic compared with plasma (Table 1). While its accessibility and cost-effectiveness have made LRS the most commonly studied alternative to Plasma-Lyte A, its presence of calcium raises concern for potential calcium influx during ischaemia-reperfusion, and its slight hypotonicity may lead to cell swelling, potentially compromising myocardial integrity. Despite these theoretical concerns, LRS also provides beneficial features. The lactate component functions as an endogenous buffer that is metabolized to bicarbonate in the liver, supporting acid-base homeostasis. Clinical trials, including randomized controlled studies, have demonstrated that LRS-based del Nido cardioplegia offers comparable myocardial protection and early postoperative outcomes to the standard Plasma-Lyte formulation in adult cardiac surgery.

Over the past decade, accumulating clinical data—including our own series—have progressively established LRS as a safe and effective base for del Nido cardioplegia. We first reported, in 2017, the initial Thai experience using LRS as a substitute for Plasma-Lyte A in 35 mixed adult and congenital cardiac operations. A single-dose regimen (20 mL/kg, 1:4 blood-to-crystalloid ratio) achieved reliable cardiac arrest with low incidences of ventricular fibrillation (11%) and new-onset atrial fibrillation (17%), and the observed mortality matched the predicted Society of Thoracic Surgeons risk score, confirming early safety and feasibility.^13^ We subsequently demonstrated, in 2019, that LRS-based del Nido cardioplegia provided similar or superior myocardial protection compared with conventional blood cardioplegia in adult cardiac surgery. The LRS group exhibited significantly lower troponin-T release, a markedly smaller total cardioplegia volume, fewer doses, and a lower incidence of ventricular fibrillation. Patients who received LRS-based del Nido cardioplegia also had shorter ICU and hospital stays, reduced vasopressor and inotropic support duration, and a lower incidence of postoperative atrial fibrillation or flutter, indicating enhanced perioperative efficiency and faster recovery.^14^ In 2023, we compared LRS-based del Nido cardioplegia with histidine-tryptophan-ketoglutarate (HTK) solution in patients undergoing valvular surgery and found equivalent troponin-T release but clear clinical advantages in the LRS group, including a lower incidence of ventricular fibrillation, smaller cardioplegia volume, shorter hospitalization, and reduced transfusion requirements.^15^ Our most recent randomized controlled trial in 2024, enrolling 200 adults undergoing elective CABG or valve surgery, confirmed the equivalence of LRS-based del Nido cardioplegia relative to the original Plasma-Lyte A formulation: postoperative troponin-T at 24 hours and all major clinical end-points—including ejection fraction, inotrope use, arrhythmia, ICU stay, and mortality—were statistically indistinguishable despite the lower pH and higher calcium content of the LRS mixture.^16^

Complementary paediatric evidence from Sithiamnuai et al further supports the safety and efficacy of LRS-based del Nido cardioplegia in congenital heart surgery. The authors evaluated 116 consecutive paediatric patients who underwent congenital cardiac operations requiring cardioplegic arrest. Postoperative parameters—including intubation duration, ICU stay, and vasoactive medication requirements immediately and 24 hours after surgery—were not significantly different between groups. Notably, the del Nido group required less blood transfusion, and the incidence of postoperative complications was comparable. These findings confirm that clinical outcomes of LRS-based modified del Nido cardioplegia are equivalent to those achieved with conventional blood cardioplegia, supporting its applicability and safety in paediatric patients.^17^

Kumar and colleagues conducted an observational study of 88 adults undergoing elective cardiac surgery to compare LRS-based del Nido cardioplegia with the standard Plasma-Lyte A formulation. The LRS group showed higher coronary sinus lactate immediately after reperfusion and troponin I levels at 24 hours. However, secondary outcomes—including ejection fraction, cross-clamp and bypass times, inotropic scores, and ICU stay—did not differ between groups. Although no clinical disadvantage was observed, the authors advised caution in using LRS-based del Nido cardioplegia until validated by larger multicentre trials. The study was limited by its observational design, small sample size, and restricted patient population, and lacked detailed reporting of the types of procedures performed, warranting careful interpretation of its findings.^18^

Kachoueian and colleagues conducted a prospective, randomized, blinded trial involving 109 adult patients undergoing isolated CABG to compare LRS-based del Nido cardioplegia with the standard Plasma-Lyte A formulation. The LRS group exhibited significantly higher troponin I levels both on ICU admission and at 24 hours, as well as a greater requirement for intraoperative epinephrine. However, there were no significant differences in postoperative ejection fraction, cardiopulmonary bypass or cross-clamp durations, arrhythmia incidence, transfusion needs, ICU stay, or mortality between groups. The authors recommended the continued use of standard Plasma-Lyte A-based del Nido cardioplegia for CABG surgery because of its potentially better biochemical myocardial protection. This study should be interpreted with caution due to its small sample size and the absence of a clearly defined primary end-point, as the sample size calculation was based on ICU stay rather than biochemical markers of myocardial injury.^19^

Ali and colleagues conducted a randomized, double-blind controlled trial involving 120 adult patients undergoing isolated CABG to compare 3 crystalloid base solutions for del Nido cardioplegia: NSS, LRS, and plain Ringer. The investigators found no significant intergroup differences in cardiac biomarkers (troponin T, troponin I, CK-MB, or lactate) at any time point. Likewise, there were no significant differences in postoperative arrhythmia, ejection fraction, ICU or hospital stay, or mortality among the 3 groups. The authors concluded that Normal Saline, lactated Ringer’s, and plain Ringer are equally effective base solutions for del Nido cardioplegia, providing comparable myocardial protection and clinical outcomes.^11^

In summary, the collective evidence from multicentre studies and randomized trials indicates that LRS-based del Nido cardioplegia provides safe, effective, and clinically equivalent myocardial protection to Plasma-Lyte A in both adult and paediatric cardiac surgery. Although minor biochemical differences have been observed, these have not translated into adverse outcomes. Lactated Ringer’s solution therefore represents a cost-effective, physiologic, and globally accessible alternative for del Nido cardioplegia, particularly valuable in resource-limited settings where Plasma-Lyte A is unavailable.

Given ongoing interest in balanced crystalloid alternatives, other solutions with similar electrolyte composition have also been considered as potential substitutes for Plasma-Lyte A. Ringer’s acetate, which shares an electrolyte profile similar to LRS but contains acetate as the primary buffer (Table 1), may also represent a theoretically suitable alternative base solution; however, no published data to date have evaluated its use in del Nido cardioplegia.

## PLAIN RINGER’S SOLUTION

Plain Ringer’s solution (also known as Ringer’s injection) is an isotonic crystalloid composed of sodium, chloride, potassium, and calcium ions, but without any buffer (eg, lactate, acetate, or gluconate). Its approximate composition per litre is described in Table 1. Because of its high chloride concentration and absence of a buffer, large-volume administration can cause hyperchloremic metabolic acidosis. The solution is physiologic in ionic content but lacks mechanisms for acid-base equilibrium. Slightly hypertonic nature may cause cell shrinkage, potentially limiting intracellular oedema but risking mild cellular dehydration. The presence of calcium supports a physiologic ionic milieu but may increase the risk of intracellular Ca^2+^ influx during reperfusion. Clinical evaluation of plain Ringer-based del Nido cardioplegia has been reported in both adult and paediatric populations.

In a prospective, double-blind randomized controlled trial, Talwar et al evaluated the safety and efficacy of using plain Ringer as the base solution for modified del Nido cardioplegia compared with Plasma-Lyte A in 80 paediatric patients (<12 years) undergoing intracardiac repair of Tetralogy of Fallot. The primary end-point, cardiac index, was comparable between groups, confirming the noninferiority of the Plain Ringer-based solution. Furthermore, there were no significant differences in ventricular arrhythmias following aortic cross-clamp release, postoperative inotropic score, duration of mechanical ventilation, ICU or hospital stay, or in biochemical and structural markers of myocardial injury, including IL-6, TNF-α, troponin-I, and electron microscopy findings. These results demonstrate that the plain Ringer-based modified del Nido cardioplegia provides equivalent myocardial protection to the standard Plasma-Lyte-based formulation. The authors concluded that this alternative is safe, effective, and cost-efficient, representing a practical option for developing regions where Plasma-Lyte A is either unavailable or prohibitively expensive.^20^

As mentioned previously, in a double-blind randomized trial involving 120 adults undergoing elective isolated CABG, Ali et al compared NSS-based, LRS-based, and plain Ringer-based del Nido cardioplegia. Serum CK-MB, troponin T, troponin I, and lactate levels measured at 2, 12, and 24 hours postoperatively showed no significant differences among groups. Secondary outcomes—including aortic cross-clamp time, ventricular fibrillation, atrial fibrillation, duration of mechanical ventilation, and ICU stay—were also comparable, indicating equivalent myocardial protection across all base solutions.^11^

In summary, these findings support plain Ringer as a safe, effective, and economical alternative for del Nido cardioplegia, particularly valuable in resource-limited settings where Plasma-Lyte A is unavailable. Although it lacks buffering capacity and contains calcium, bicarbonate supplementation and balanced additives can effectively mitigate these limitations.

## ISOLYTE S

Isolyte S (B. Braun, global manufacturer) is a balanced, calcium-free, isotonic multiple-electrolyte solution designed to closely mimic the ionic and acid-base composition of human plasma. Its buffering system contains acetate and gluconate, both metabolized to bicarbonate, thereby maintaining physiologic pH without lactate accumulation. Functionally equivalent to Plasma-Lyte A, Isolyte S provides a plasma-like electrolyte profile with slight variations in buffer concentrations. The small amount of phosphate in Isolyte S (∼1 mEq/L) functions primarily as a minor physiologic buffer and metabolic substrate, supporting acid-base stability and cellular energetics without affecting calcium compatibility. As a physiologic, calcium-free crystalloid, it serves both as an intravenous replacement fluid and an ideal base solution for del Nido cardioplegia, offering stable ionic balance, effective buffering capacity, and compatibility with blood products (Table 1).

Karaarslan et al compared modified del Nido cardioplegia (4:1 blood-to-Isolyte S ratio) with the classical del Nido formulation (1:4 ratio) in 70 patients undergoing isolated CABG. Both used Isolyte S as the base solution. The modified group showed significantly lower fibrillation and defibrillation rates, shorter cardiac restart time, less postoperative hemodilution, and reduced transfusion requirements, with no differences in cross-clamp time, enzyme markers, or mortality. These findings indicate that Isolyte S-based modified del Nido cardioplegia provides equivalent myocardial protection to the classical formula while improving electrical stability and minimizing blood loss in adult CABG patients.^21^

Erol et al evaluated Isolyte S-based modified del Nido cardioplegia in 123 patients undergoing CABG with an ejection fraction ≤35%. Compared with conventional blood cardioplegia, the Isolyte S group demonstrated lower intraoperative defibrillation, reduced inotrope requirement, lower postoperative troponin-I at 12 hours, and less postoperative atrial fibrillation, with no differences in cross-clamp duration, ICU stay, or mortality. These findings indicate that Isolyte S-based del Nido cardioplegia provides myocardial protection equivalent to blood cardioplegia while potentially improving electrical stability and metabolic recovery in patients with impaired ventricular function.^22^ In conjunction with previous studies using Plasma-Lyte-based del Nido formulations that report comparable safety and efficacy in adult cardiac surgery, this evidence supports Isolyte S as a physiologically appropriate and clinically effective alternative base solution for del Nido cardioplegia, particularly valuable where Plasma-Lyte A is not readily available.

## IONOSTERIL

Ionosteril (Fresenius Medical Care, Schweinfurt, Germany) is a balanced, multiple-electrolyte infusion, designed to mimic plasma composition and maintain acid-base equilibrium. It contains sodium 137 mmol/L, potassium 4 mmol/L, magnesium 1.25 mmol/L, chloride 110 mmol/L, acetate 27 mmol/L, gluconate 23 mmol/L, and calcium 1.65 mmol/L, with a physiologic pH of 7.4 and osmolarity ≈ 291 mOsm/L (Table 1). These features make it isotonic and alkalinizing through metabolizable anions (acetate/gluconate) while providing magnesium for membrane stabilization. Compared with Plasma-Lyte A—the original del Nido base—it contains slightly higher calcium but otherwise similar ionic balance, thus serving as an excellent substitute where Plasma-Lyte A is unavailable.

Supporting evidence comes from Kang et al, who compared Ionosteril-based modified del Nido cardioplegia with Custodiol (HTK, Dr Franz Köhler Chemie, Bensheim, Germany) in 355 patients undergoing isolated minimally invasive mitral valve repair. After propensity-score matching of 156 patient pairs, the Ionosteril-based del Nido group demonstrated significantly lower postoperative CK and CK-MB levels, similar troponin T concentrations, and lower lactate levels, indicating effective myocardial preservation and reduced ischaemic stress. Custodiol, in contrast, was associated with marked intraoperative and postoperative hyponatremia, while other clinical outcomes—including arrhythmias, renal function, and mortality—were comparable between groups. Although the hospital stay was modestly longer in the del Nido group, overall results confirmed that Ionosteril-based del Nido cardioplegia provides safe, physiologic, and effective myocardial protection equivalent to Custodiol, particularly suitable for procedures with cross-clamp times up to approximately 90 minutes.^23^

## WHOLE BLOOD

Whole-blood del Nido cardioplegia is a modified formulation in which the crystalloid component of the original del Nido solution is completely replaced with oxygenated autologous blood from the cardiopulmonary bypass circuit. This adaptation enhances the physiologic properties of the solution by providing superior buffering capacity, oxygen and substrate delivery, and maintenance of oncotic pressure while minimizing hemodilution and myocardial oedema. The additives—potassium chloride, sodium bicarbonate, magnesium sulfate, mannitol, and lidocaine—are mixed directly into whole blood and delivered as a single cold dose, achieving rapid depolarizing arrest and prolonged myocardial protection for up to 90 minutes.

Clinical evidence in adult cardiac surgery, particularly the retrospective analysis by Haider et al, supports these advantages. In that study of 234 patients undergoing elective CABG, those who received Whole-blood del Nido cardioplegia (n = 114) were compared with patients who received conventional diluted del Nido cardioplegia (blood: crystalloid = 1:4; n = 120). The Whole-blood del Nido cardioplegia group exhibited significantly less hemodilution, reflected by higher peri- and post-bypass hemoglobin levels, and showed lower myocardial injury markers, with markedly reduced peak troponin-T and CK-MB levels. Whole-blood del Nido cardioplegia also provided better renal protection, evidenced by lower postoperative serum creatinine and blood urea levels, and required less inotropic support, with a significantly lower adrenaline infusion rate. Hospital stay was shorter, while rates of arrhythmia, transfusion, and mortality were similar between groups, confirming its safety.^24^

More recently, Brown et al evaluated 272 patients with preoperative left ventricular ejection fraction ≤40% who underwent isolated CABG using either conventional cardioplegia or a modified whole-blood del Nido solution. Despite the higher-risk population, outcomes—including 30-day mortality, arrhythmia, ICU stay, and total hospitalization—were equivalent between groups, while the whole-blood del Nido cardioplegia group had shorter bypass times and higher postoperative hemoglobin levels, confirming reduced hemodilution and preserved myocardial protection even in low-EF patients.^25^

Collectively, these findings demonstrate that whole-blood del Nido cardioplegia offers effective myocardial protection, improved metabolic and renal stability, and operational simplicity, with physiologic and economic advantages over diluted or crystalloid-based formulations in adult cardiac surgery.

## FUTURE DIRECTIONS

Although del Nido cardioplegia has been successfully adapted for use with multiple base solutions, several important questions remain. Future research should focus on standardizing formulations of modified del Nido cardioplegia to ensure consistent ionic composition, buffering capacity, and stability across institutions and compounding pharmacies. Comparative multicentre randomized controlled trials are needed to confirm the biochemical and clinical equivalence of alternative base solutions against the original Plasma-Lyte A formulation in both adult and paediatric populations.

A critical additional gap in current knowledge is the lack of data on the final delivered ionic composition of del Nido cardioplegia after complete mixing with its additives and blood component. Systematic analyses of final electrolyte concentrations, buffering systems, osmolarity, and calcium availability across different base solutions are essential to identify the key physiologic determinants of myocardial protection. Addressing this gap would help define which components of the final mixture most strongly influence clinical outcomes.

Further studies should also evaluate the long-term outcomes of patients receiving alternative base solutions, including ventricular recovery, arrhythmia burden, and postoperative organ function. Pharmaceutical and stability analyses are warranted to establish validated shelf lives, sterility assurance, and compatibility with storage and transport conditions. Finally, translational research exploring personalized cardioplegia strategies—tailoring electrolyte and substrate composition to specific patient factors such as age, ventricular function, ischaemic time, or comorbidity—may further refine myocardial protection and expand the versatility of the del Nido technique.

## CONCLUSIONS

The evolution of del Nido cardioplegia from a paediatric innovation at Boston Children’s Hospital to a globally adopted strategy for adult cardiac surgery reflects its simplicity, reproducibility, and reliable myocardial protection. Although the original formulation using Plasma-Lyte A remains the standard reference, increasing attention has focused on identifying alternative base solutions that maintain physiologic balance, reduce cost, and improve accessibility.

Cumulative experimental and clinical evidence demonstrates that modified del Nido cardioplegia prepared with whole blood, lactated Ringer’s, Normal Saline, plain Ringer’s, Isolyte S, or Ionosteril can provide comparable myocardial protection and clinical outcomes when appropriately buffered and supplemented. Each alternative possesses unique physiochemical characteristics—such as calcium content, tonicity, and buffering capacity—that influence myocardial physiology during ischaemia and reperfusion.

The choice of base solution should therefore be guided by local availability, cost, and compounding capability rather than adherence to a single proprietary formulation. In resource-limited settings, these alternatives enable broader and safer implementation of del Nido cardioplegia without compromising myocardial protection. Future multicentre randomized trials and standardized stability assessments are warranted to define optimal formulations and ensure consistent quality across clinical practice worldwide.

## Data Availability

The data underlying this article will be shared on reasonable request to the corresponding author.
